# Prebiotic effect during the first year of life in healthy infants fed formula containing GOS as the only prebiotic: a multicentre, randomised, double-blind and placebo-controlled trial

**DOI:** 10.1007/s00394-014-0689-9

**Published:** 2014-03-27

**Authors:** Carlos Sierra, María-José Bernal, Javier Blasco, Rosario Martínez, Jaime Dalmau, Inmaculada Ortuño, Beatriz Espín, María-Isabel Vasallo, David Gil, María-Luisa Vidal, Dámaso Infante, Rosaura Leis, José Maldonado, José-Manuel Moreno, Enriqueta Román

**Affiliations:** 1 Hospital Materno-Infantil, Avda. Arroyo de los Ángeles s/n, 29011 Málaga, Spain; 2 Research and Development Department, Hero Spain S.A., Avda. Murcia 1, 30820 Alcantarilla, Murcia Spain; 3 Hospital La Fe, Avda. Pianista Martínez Carrasco, s/n, 46026 Valencia, Spain; 4 Hospital Virgen del Rocío, Avda. Manuel Siurot, s/n, 41013 Sevilla, Spain; 5 Hospital Virgen de la Arrixaca, Ctra. Madrid-Cartagena, s/n, 30120 El Palmar, Murcia Spain; 6 Hospital Vall d’Hebron, Passeig Vall d’Hebron, 119-129, 08035 Barcelona, Spain; 7 Hospital Clínico Universitario, Travesía de Choupana, s/n, 15706 Santiago de Compostela, Spain; 8 Hospital Virgen de las Nieves, Avda. de las Fuerzas Armadas, 2, 18014 Granada, Spain; 9 Hospital Doce de Octubre, Avda. de Córdoba, s/n, 28041 Madrid, Spain; 10 Hospital Puerta de Hierro, Calle Manuel de Falla, 1, 28222 Majadahonda, Madrid Spain

**Keywords:** Clinical trial, Prebiotics, Infant nutrition, Infant formula, Randomised double-blind, placebo-controlled study

## Abstract

**Purpose:**

Currently, there is no consensus concerning the possible beneficial colonic and systemic effects of prebiotic-containing infant formula. This study assesses whether the feeding of a galactooligosaccharides (GOS)-containing infant formula (0.44 g/dl of GOS) and the subsequent feeding of a GOS-containing follow-on formula (0.50 g/dl of GOS) have a prebiotic effect on intestinal microbiota that helps to decrease infections and allergy manifestations in healthy infants during the first year of life.

**Methods:**

A multicentre, randomised, double-blind and placebo-controlled trial was carried out on 365 healthy term infants enrolled before 8 weeks of age and randomly assigned to a formula with or without GOS, until 12 months of age. The incidence of infections and allergy manifestations, the antibiotics prescribed and faecal characteristics were recorded up to 12 months of age, while faecal samples were collected up to 4 months for the measurement of secretory immunoglobulin A, short-chain fatty acids and microbiota.

**Results:**

A prebiotic effect on the faecal analysis was observed at 4 months of life. The GOS group showed a lower faecal pH (*P* = 0.019), a lower decreasing trend in secretory immunoglobulin A (*P* = 0.078), lower butyric acid concentration (*P* = 0.040) and an increase in *Bifidobacterium* counts (*P* = 0.010). Changes in faecal characteristics involved greater frequency (*P* < 0.001) and softer consistency (*P* < 0.05). The incidence of infections or allergic manifestations during the first year of life was similar in both groups, with no statistical differences (*P* > 0.05).

**Conclusions:**

The feeding of GOS-containing infant formula produced a definite prebiotic effect consisting of changes in faecal composition and microbiota, and in faecal consistency and the frequency of defaecation. No changes in the incidence of infection or allergic manifestation during the first year of life were observed.

## Introduction

Human milk oligosaccharides (HMOs), a complex mixture of glycan compounds, have been associated with the prevention of intestinal diseases, which are partially mediated by the modulation of the intestinal microbial ecology and immunological homeostasis [1, 2]. Prebiotics are non-digestible carbohydrates similar to HMOs. Because HMOs include a high quantity of galactose, galactooligosaccharides (GOS) are often the predominant prebiotic oligosaccharides used to supplement infant formulas. The prebiotic effect is defined as “the selective stimulation of growth and/or activity(ies) of one or a limited number of microbial genus(era)/species in the gut microbiota that confer(s) health benefits to the host” [3]. The main health benefits that intestinal microbiota confer are an improvement in faeces quality (pH, short-chain fatty acid content, frequency and consistency), a reduction in the risk of gastrointestinal infections, improved general well-being and a reduced incidence of allergic symptoms such as atopic eczema [3]. Infant formulas with added prebiotics are also associated with beneficial effects, but few studies have demonstrated an improvement in clinical outcomes such as infections and allergic manifestations [4–8]. Consequently, recent systematic reviews have concluded that further evidence is needed in order to recommend or not the routine addition of prebiotics to infant formulas to prevent infections [9] and allergy [9, 10]. The inclusion of GOS in infant formulas is believed to lead to an intestinal microbiota composition, with more bifidobacteria, fewer pathogens and a metabolic activity similar to that described in breastfed infants [11], resulting in beneficial effects on health through immune system regulation [3, 12]. No published studies have evaluated the effect of GOS-containing infant formulas on infections and allergy. Only prebiotic effects on the growth of *Bifidobacterium* and *Lactobacillus* genera have been evaluated [13, 14].

Our hypothesis was that the feeding of a GOS-containing formula would have a prebiotic effect on intestinal microbiota, which might help to reduce infections and allergic manifestations. To test this hypothesis, we conducted a prospective, randomised, double-blind and placebo-controlled trial to assess the effects of prebiotic supplementation on intestinal microbiota and some related parameters in the colon and on the incidence of infectious episodes and allergic manifestations during the first year of life.

## Methods and materials

### Study design and protocol

Healthy term infants of <2 months of age were recruited for a multicentre, randomised, double-blind and placebo-controlled trial in eight Spanish hospitals: Hospital Materno-Infantil (Málaga), Hospital Materno-Infantil (Granada), Hospital Virgen del Rocío (Sevilla), Hospital Virgen de la Arrixaca (Murcia), Hospital Infantil La Fe (Valencia), Hospital Doce de Octubre (Madrid), Hospital Clínico Universitario (Santiago de Compostela) and Hospital Infantil Vall d’Hebrón (Barcelona). The study was conducted according to the guidelines established in the Declaration of Helsinki. The protocol was approved by the ethics committees at each hospital, and written informed parental consent was obtained for each infant before inclusion.

Eligible infants had a gestational age of 37–42 weeks and a birth weight greater than 2,500 g and were exclusively formula fed for at least 15 days prior to enrolment. Infants that had previously consumed either prebiotics or probiotics and infants who themselves, or their mothers, had clinically significant diseases were excluded.

Infants were randomly assigned to receive an infant formula until 6 months of age and then the follow-on formula until 12 months of age either with GOS supplementation (GOS group) or without (control group). Enrolment and randomization were performed before or at 2 months of age. A separate randomization schedule in blocks of 10 was prepared for each study site by an external independent agent and provided in consecutively numbered and sealed envelopes. The introduction of complementary feeding was allowed at 4 months. Prebiotics and probiotics were not allowed during the feeding period. The contents of GOS were 0.44 g/dl in the study infant formula and 0.50 g/dl in the study follow-on formula (commercially available as Nutradefense^®^ infant and follow-on formulas) (Table 1). Both infant and follow-on formulas were provided in powdered form, had identical sensorial characteristics and the same label and were designed, produced, codified (two numbers printed on cans) and provided by the Hero Group. Both the investigators and the infants’ parents were blind to the group allocation. The blinding was broken once the statistical analysis was completed.

**Table 1:** Nutritional composition of the four formulas used in the study per dl

	Infant formula		Follow-on formula	
	Control	GOS	Control	GOS
Energy (kcal)	67	65	66	70
Protein (g)	1.4	1.4	1.6	1.6
Casein–whey	40:60	40:60	50:50	50:50
Lactose (g)	6.98	6.70	5.2	5.9
Maltodextrins (g)	0.72	0.80	3.2	3.2
GOS (g)	–	0.44	–	0.50
Fat (g)	3.5	3.5	2.9	2.9
Linoleic acid (mg)	442	442	433	353
α-linolenic acid (mg)	62	62	60.8	50.3
Arachidonic acid (mg)	6.9	6.9	2.7	2.7
Docosahexaenoic acid (mg)	6.9	6.9	2.7	2.7
Nucleotides (mg)	3.3	3.3	3.4	3.4

### Study outcomes and data collection

Acute diarrhoea was defined as semiliquid or liquid faeces in three or more depositions per day for at least 3 days. Upper respiratory tract infections (URTI) were diagnosed by the primary care paediatrician based on specific symptoms. Recurrent URTI were defined as more than 3 episodes in 12 months. Any sign or symptom related to allergy, wheezing and atopic dermatitis (AD) and infection (fever, cough, rhinitis and diarrhoea) was recorded and closely supervised by the paediatrician. Atopic sensitisation to cow’s milk, egg and fish was evaluated by skin prick testing (SPT) at the age of 12 months.

For 5 days before each visit, parents recorded the frequency of defaecation, faecal consistency and intake of formula in daily records. Faecal consistency was rated on a 1–5 scale (1 = watery, 2 = loose, 3 = lumpy, 4 = soft, 5 = hard).

All parameters (URTI, wheezing, AD, fever, cough, rhinitis, diarrhoea, frequency of defaecation, faecal consistency and intake of formula) were recorded (reported by the parents and reviewed by a primary care paediatrician) during the visits at 3 (75–99 days), 4 (107–142 days), 6 (168–199 days), 9 (261–289 days) and 12 (352–375 days) months of life. Anthropometric measurements (weight, length and head circumference) were taken by the paediatrician at enrolment and during the visits.

To study the possible prebiotic effect, fresh faecal samples were collected from nappies in a plastic faeces container, frozen immediately after collection by the parents at home and stored until they were taken to a central collection site. Faeces were then kept at −80 °C until analysis. The faecal samples were collected in a subgroup of infants at enrolment and at 4 months of age, and pH, secretory immunoglobulin A (sIgA), short-chain fatty acids (SCFAs) and microbiota parameters were measured.

To measure faecal pH, an aliquot of 1 g of faeces was diluted tenfold in Milli-Q water. After dilution, the faecal pH was measured with a GLP21 pH meter (Crison, Barcelona, Spain). Faecal sIgA concentration was measured with the Immunodiagnostik K8870 (Bensheim, Germany) commercial enzyme-linked immunosorbent assay, according to the manufacturer’s instructions. SCFAs were detected and quantified using gas chromatography, as previously described [15].

Before bacterial quantification by real-time-polymerase chain reactions (PCRs), the genomic DNA in the faecal samples and the DNA from bacterial cultures used for calibration curves were extracted using a QIAamp DNA stool mini kit (Qiagen, Hilden, Germany) [16]. PCRs were conducted on a 25-μl final volume with 2–3 μl of template and 0.5 μM to 0.7 μM of primer pairs corresponding to each bacterium: *Bifidobacterium* [17], *Lactobacillus* [18], *Bacteroides* [19], *Bifidobacterium species* [20] and *Clostridium difficile* [21]. Bacterial counts were expressed as log_10_ colony-forming units (CFU) per gram of faecal samples.

### Statistical analysis

The power of the study was calculated considering respiratory infections and *Bifidobacterium* population as primary outcomes. Sample size was estimated to detect a 20 % reduction in the incidence of respiratory infections, considering that each infant would have on average 6.3 episodes [mean 5.1, interquartile range (IQR) 3.3–7.8] of URTI in the first year of life [22]. Thus, with the power set at 85 % and the significance level at 0.05, the required sample size was about 156 infants per each cohort to show a difference of 20 % between the groups. Given that the variability of respiratory infections is greater than gastrointestinal infections, this sample size would also be sufficient for detecting differences in gastrointestinal infections. For the evaluation of the prebiotic effect, the minimum number of subjects, based on the faecal concentration of *Bifidobacterium*, was 20 subjects per group to achieve a mean difference of 30 % compared with the control formula [23] and taking into account 20 % of dropouts, with a probability of 80 % and a significance level of 0.05.

Continuous variables are reported as the mean and standard deviation (SD), or median and IQR, and categorical variables are reported as numbers or percentages. To detect differences at enrolment between the feeding groups in each quantitative variable, an unpaired student’s *t* test was used; for variables expressed as percentages, a Pearson Chi-square test was used. The results for the two groups were evaluated with an unpaired student’s* t* test for anthropometric measurements and for the incidence of diarrhoea, URTI and allergic manifestations. To compare the proportion of infants with URTI and acute diarrhoea incidences, as well as the antibiotic prescription rates of both groups, a Pearson Chi-square test was used. All parameters of the collected faecal samples were analysed with a repeated-measures analysis of variance. Pearson’s correlation coefficient was calculated among all parameters analysed in faeces. A general linear univariate model was used to analyse frequency of defaecation, and a Pearson Chi-square test was used to analyse faecal consistency.

All results with a significance level of *P* < 0.05 were considered statistically significant. Statistical analyses of data were performed using the Statistical Package for the Social Sciences (SPSS Version 18.0; Inc., Chicago, IL, USA).

## Results

### Subjects

**Fig. 1:**
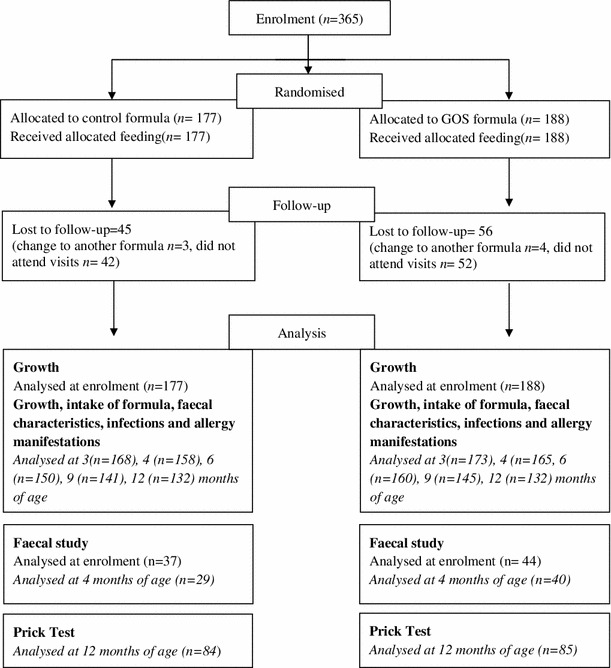
Flow chart of infants enrolled and disposition of the subjects throughout the study

A total of 365 infants were enrolled and randomized in the study, 177 in the control group and 188 in the GOS group (Fig. 1). The percentage of dropouts was similar in both feeding groups at around 27 %. One hundred and thirty-two infants in each group successfully completed the study (Fig. 1). At enrolment, there were no differences between the two groups in terms of gender, gestational age, type of delivery, birth weight, age at inclusion, number of days with exclusive breastfeeding before enrolment, family history of atopy, smoking parents and furry pets at home (Table 2).

**Table 2:** Characteristics of infants enrolled in the study

	Control group (*n* = 177)	GOS group (*n* = 188)	*P* value^a^
Male/female	96/81	107/81	0.673
Gestational age (week) (mean ± SD)	39.31 ± 1.20	39.13 ± 1.36	0.189
Delivery (vaginal/caesarean)	122/55	136/55	0.474
Birth weight(g) (mean ± SD)	3,249 ± 426	3,222 ± 488	0.570
Age at enrolment (days) (mean ± SD)	32.16 ± 18.21	29.38 ± 17.74	0.140
Exclusive breastfeeding before enrolment (days) (mean ± SD)	19.54 ± 15.44	16.82 ± 14.92	0.300
Family history of atopy			
Father (%)	17 ± 10	20 ± 11	0.744
Mother (%)	25 ± 14	29 ± 15	0.726
One brother (%)	7 ± 4	13 ± 7	0.214
Two brothers (%)	4 ± 2	5 ± 3	0.586
Smoking parents (%)	60 ± 34	74 ± 39	0.279
Furry pets at home (%)	22 ± 12	34 ± 18	0.134

^a^Difference between the groups tested with unpaired Student’s *t* test for variables expressed as mean ± SD and a Pearson Chi-square for variables expressed as percentage

During the feeding period, all infants had adequate growth (e.g. the mean weight at 12 months was 9.94 ± 1.37 kg for the control group and 9.98 ± 1.10 kg for the GOS group) and the intake of formula appropriate for age, with no significant differences between the feeding groups at 3, 4, 6, 9 and 12 months of age (data not shown). No issues related to consumption of the formula (regurgitations, gassiness or rejections) were reported.

At enrolment, the faecal subsample included 81 infants (control group, 37; GOS group, 44); 12 were excluded due to parental decision, the introduction of a non-study formula, or improper faecal sample delivery or preservation. At 4 months, 69 participants were included in the subset analysis (control group, 29; GOS group, 40) (Fig. 1).

### Faecal analysis

The results of the faecal parameters at enrolment and at 4 months of age in both feeding groups are shown in Tables 3, 4 and 5. No significant differences in faecal parameters were found between the groups at enrolment.

**Table 3:** Results for faecal pH, sIgA and SCFAs, expressed as mean ± SD and median [IQR]

	Control group		GOS group		*P* value^a^
	Enrolment (*n* = 37)	4 months (*n* = 29)	Enrolment (*n* = 44)	4 months n = 40	
pH	6.63 ± 0.89	7.09 ± 0.69	6.37 ± 0.94	6.57 ± 0.82	0.019
sIgA (mg/g)	3.45 ± 4.45	1.72 ± 1.46	4.35 ± 5.41	2.76 ± 2.06	0.078
	1.84 [0.56–3.94]	1.12 [0.63–2.68	2.36 [0.87–4.82]	2.32 [1.31–3.54]	
Total SCFAs (µmol/g)	67.22 ± 26.46	98.91 ± 39.56	74.95 ± 40.13	93.36 ± 40.70	0.567
Acetic acid (%)^b^	78.13 ± 12.58	75.72 ± 9.76	78.48 ± 33.90	84.77 ± 8.52	0.005
Propionic (%)^b^	15.49 ± 10.14	15.27 ± 6.30	11.45 ± 8.91	11.53 ± 6.62	0.015
Butyric acid (%)^b^	4.01 ± 6.99	5.59 ± 4.06	4.73 ± 6.61	2.29 ± 2.29	0.040

^a^Differences between the groups over time were analysed with a repeated-measures analysis of variance

^b^Results expressed as percentage of total SCFAs

**Table 4:** Intestinal microbiota counts in faecal samples of infants (log_10_ CFU/g)

	Control group		GOS group		*P* value^a^
	Enrolment	4 months	Enrolment	4 months	
*Bifidobacterium* spp	8.22 ± 1.53	8.02 ± 1.66	7.78 ± 1.78	8.65 ± 1.31	Δ_I_ = 0.010
*Lactobacillus* spp	6.72 ± 0.70	6.87 ± 0.61	6.62 ± 0.75	7.11 ± 0.89	Δ_I_ = 0.151
*Bacteroides* spp	6.39 ± 2.27	6.83 ± 2.17	6.07 ± 2,10	7.12 ± 2.04	Δ_I_ = 0.187
*Clostridium difficile*	4.45 ± 1.43	5.64 ± 1.34	5.10 ± 0.84	5.79 ± 0.91	Δ_I_ = 0.065

Results are expressed as the mean ± SD

^a^Differences between the change from enrolment to month 4 (Δ_I_) of both feeding groups tested with a unpaired Student’s *t* test

**Table 5:** *Bifidobacterium* species at enrolment and at 4 months of age, as bacterial counts (log_10_ CFU/g fresh stools) and percentage of colonised infants

	Control group		GOS group		*P* value^a^
	Enrolment	4 months	Enrolment	4 months	
*B. bifidum*	6.48 ± 1.45 72.4 %	7.16 ± 1.47 82.8 %	6.99 ± 1.45 72.5 %	7.94 ± 1.90 90.0 %	0.082
*B. longum*	8.72 ± 0.96 75.9 %	8.96 ± 0.86 75.9 %	8.56 ± 1.39 77.5 %	9.43 ± 0.57 77.5 %	0.310
*B. adolescentis*	4.71 ± 0.57 58.6 %	5.41 ± 1.19 62.1 %	5.57 ± 1.46 50.0 %	5.64 ± 1.51 52.5 %	0.079
*B. breve*	5.36 ± 2.05 27.6 %	4.93 ± 1.44 41.4 %^b^	5.78 ± 1.59 32.5 %	6.37 ± 1.57 57.5 %^b^	0.055
*B. dentium*	4.30 ± 0.76 20.7 %	4.99 ± 0.24 20.7 %	4.95 ± 1.37 25.0 %	5.68 ± 1.44 15.0 %	0.410
*B. catenulatum*	6.26 ± 0.50 24.1 %	6.70 ± 0.70 20.7 %	6.75 ± 0.81 17.5 %	7.09 ± 0.59 27.5 %	0.094

Results expressed as the mean ± SD

^a^Differences between the feeding groups using unpaired Student’s *t* test

^b^Significant differences (*P* < 0.05) between the feeding groups at 4 months for percentage of colonised infants using Pearson Chi-square test

Faecal pH was significantly influenced by the formula received and was lower in the GOS group than in the control group (6.77 ± 0.82 vs. 7.09 ± 0.69, *P* = 0.019) (Table 3) at 4 months of age. The faecal sIgA concentration decreased during the feeding period in both groups (*P* = 0.046), although we observed no significant differences between the feeding groups at 4 months of age (control group, 1.72 ± 1.46 mg/g vs. GOS group, 2.76 ± 2.06 mg/g). A trend towards a lower reduction of faecal sIgA was observed for infants in the GOS group versus control group (*P* = 0.078) (Table 3).

A significant increase in total SCFAs (μmol/g) from enrolment through 4 months of age was observed for both feeding groups (*P* < 0.001) (Table 3). When analysed as a percentage of total SCFAs, significant group differences were observed for the three main fatty acids. The percentage of acid acetic was higher in the GOS group than in the control group (84.77 vs. 75.72 %, *P* = 0.005), while the percentages of propionic and butyric acids were lower in the GOS group compared with the control group (11.53 vs. 15.27 %, *P* = 0.015 and 2.29 vs. 5.59 %, *P* = 0.040, respectively) (Table 3). A negative correlation between pH and acetic acid (*r* = −0.630, *P* < 0.001) was observed.

For mean faecal bacterial counts (log_10_ CFU/g) from enrolment through 4 months of age, *Bifidobacterium* increased in infants in the GOS group compared with the control group (0.97 vs. −0.23; *P* = 0.010) (Table 4). No group differences were observed for *Lactobacillus* and *Bacteroides*. *C. difficile* significantly increased through 4 months of age in both feeding groups (*P* < 0.05), but was not influenced by the feeding type (*P* = 0.065) (Table 4). The percentage of infants with detectable *C. difficile* at enrolment was similar between the feeding groups (control group, 23.3 % vs. GOS group, 21.4 %). However, by 4 months of age, the percentage of infants in the GOS group with detectable *C. difficile* was significantly lower (GOS group, 45.2 % vs. control group, 63.3 %; *P* = 0.037).

The characterisation of *Bifidobacterium* species revealed that the percentage of infants colonised by *B. breve* and *B. adolescentis* increased from enrolment through 4 months of age. The percentage of *B. breve*-colonised infants at enrolment was similar between the feeding groups (control group, 27.5 % vs. GOS group, 32.5 %). However, at 4 months of age, percentage of infants in the GOS group with detectable *B. breve* was significantly higher (control group, 41.4 % vs. GOS group, 57.5 %, *P* < 0.05) (Table 5). Moreover, the *B. breve* count increased more strongly in the GOS group (6.37 ± 1.57 log_10_ CFU/g) than in the control group (4.93 ± 1.44 log_10_ CFU/g) (*P* = 0.055; Table 5).

The Pearson correlation was applied to determine the relationship between the microbiota and the SCFA concentration. A positive significant correlation was obtained between acetic acid and the *Bifidobacterium* population (*r* = 0.251; *P* = 0.002) and a negative correlation between butyric acid and *Bifidobacterium* (*r* = −0.235; *P* = 0.003).

### Infections and allergy

The number of URTI episodes and the number of infants with recurrent URTI were similar in both groups [odds ratio (OR) = 1.057, CI = 0.550–2.032, *P* = 0.868] (Table 6). No significant differences were found for the number of episodes of diarrhoea per infant up to 12 months of age between the feeding groups (control group, 0.20 ± 0.52 vs. GOS group, 0.27 ± 0.67, *P* = 0.355) (Table 6). No significant differences between the feeding groups in the number of infants with at least one episode of diarrhoea (control group, 15.90 % vs. GOS group 18.18 %, *P* = 0.604) were observed (Table 6). Additionally, the prescription of antibiotics did not differ significantly between the groups (control group, 19.87 % vs.GOS group 17.79 %; *P* = 0.481) (Table 6).

**Table 6:** Results of diarrhoea, URTI, antibiotic treatment, allergic manifestations and positive sensitisation skin prick test (SPT) during the first year

	Control group	GOS group	Odds ratio (95 % CI)	*P* value^a^
No. of episodes of diarrhoea/infant (mean ± SD)	0.20 ± 0.52	0.27 ± 0.67		0.355
Infants with at least 1 episode of diarrhoea/year (%)	15.9	18.2	1.185 (0.623–2.253)	0.800
No. of episodes of URTI/infant (mean ± SD)	1.65 ± 1.83	1.84 ± 2.01		0.443
Infants with at least 3 episodes of URTI/year (%)	15.9	16.7	1.057 (0.550–2.032)	0.868
Antibiotic treatment (%)	19.8	17.8	0.873 (0.598–1.274)	0.481
Allergic manifestations (AD, wheezing, food allergy)	28/132	39/132	1.558 (0.889–2.728)	0.120
Positive sensitisation SPT	9/84	6/85	0.633 (0.215–1.864)	0.403

^a^Differences between the feeding groups. A Student’s *t* test was used for the number of diarrhoea episodes and the number of URTI episodes per infant. A Pearson Chi-square test was used to compare the proportion of infants with recurrent URTI and acute diarrhoea, as well as the rates of antibiotics used, allergic manifestations and ratio of positive sensitisation SPT in both groups

No group differences in allergic manifestations were observed up to 12 months of age (control group, 28; GOS group, 39; OR = 1.558, CI 0.889–2.728; *P* = 0.120) (Table 6). Skin prick testing was performed for 169 infants (control group, 84; GOS group, 85); 3 were positive in the control group (egg, 1; cow’s milk, 2) and 6 in the GOS group (egg, 6; cow’s milk, 2; fish, 1). No group differences in allergic sensitization were observed (OR = 0.633; CI 2.15–1.864; *P* = 0.403) (Table 6).

### Faecal characteristics

Significant differences in the frequency of defaecation (no. of depositions/day) were observed between the feeding groups at 3 months of age (control group, 1.26 ± 0.83 vs. GOS group, 1.45 ± 0.97; *P* < 0.05) and at 4 months of age (control group, 1.26 ± 0.94 vs. GOS group, 1.50 ± 0.99; *P* < 0.05), while at 6, 9 and 12 months of age, the frequency of defaecation in both groups was similar. Moreover, statistically significant differences in faecal consistency were detected between the feeding groups, especially at 3, 4 and 6 months of age. In general, infants fed the GOS formula showed a higher percentage of lumpy and soft faeces and lower percentage of hard faeces compared with the infants fed the control formula (Table 7).

**Table 7:** Faecal consistency and frequency of defaecation in both feeding groups up to 12 months of age

	3 months		4 months		6 months		9 months		12 months	
	Control	GOS	Control	GOS	Control	GOS	Control	GOS	Control	GOS
Consistency number of cases (%)										
Watery	18 (3.3)	10 (1.8)	8 (1.2)	29 (4.6)*	7 (1.1)	10 (1.5)	9 (1.4)	18 (2.8)	8 (1.5)	3 (0.5)
Loose	73 (13.5)	99 (17.7)	60 (8.7)	116 (18.4)*	39 (6.0)	62 (9.1)*	34 (5.5)	70 (10.9)*	27 (5.0)	29 (5.2)
Lumpy	119 (21.9)	199 (35.7)*	117 (17.1)	211 (33.5)*	98 (15.1)	140 (20.6)*	86 (13.9)	82 (12.8)	61 (11.2)	81 (14.5)
Soft	293 (54.1)	274 (49.2)	306 (44.6)	291 (46.2)*	388 (54.7)	395 (58.0)	393 (63.5)	381 (59.7)	338 (62.6)	355 (63.5)
Hard	35 (6.4)	11 (1.97)*	53 (7.7)	32 (5.1)*	196 (30.2)	156 (23.3)*	198 (31.9)	184 (28.8)	167 (30.9)	151 (27.0)
No. of depositions/day Mean ± SD	1.26 ± 0.83	1.45 ± 0.97*	1.26 ± 0.94	1.50 ± 0.99*	1.62 ± 0.96	1.64 ± 0.98	1.82 ± 0.92	1.84 ± 0.99	1.65 ± 0.88	1.73 ± 0.89

* *P* < 0.05 differences between the feeding groups tested with an unpaired Student’s *t* test for frequency of defaecation, and a Pearson Chi-squared test for faecal consistency

## Discussion

This trial shows that supplementation of an infant/follow-on formula, with GOS as only the prebiotic, decreases intestinal pH and increases both acetic acid and the *Bifidobacterium* population. It also improves the faecal consistency and frequency of defaecation. The incidence of infections and allergy manifestations were not significantly affected.

Prebiotic substances modulate the beneficial and pathogenic intestinal microbiota and other related parameters. The clinical relevance for functional outcomes cannot be confirmed due to the low number of studies in the literature in which both intestinal and systemic changes have been studied. However, it is well established that intestinal microbiota plays a crucial role in immune system regulation [3]. The positive correlation between the *Bifidobacterium* population and the amount of intestinal sIgA is well known [24, 25]. Moreover, the intestinal pH modulates the intestinal environment, inhibiting or favouring the growth of different bacterial populations; more specifically, a decrease in intestinal pH results in a decrease in the amount of pathogenic microorganisms [26]. Consequently, it was unclear whether or not we would be able to detect the influence of the feeding of a GOS-containing infant formula on the immune system at systemic level as regard the incidence of infections and allergy manifestations through the modulation of intestinal microbiota and related biomarkers in healthy infants.

The current study demonstrated changes of several parameters in the colon. The faecal pH in the GOS group was lower than in the control group, as has been observed in other studies involving GOS [13] and GOS/fructooligosaccharides (FOS) [27, 28]. The main regulatory variable of the luminal pH is the production of SCFAs, which are fermentation products of anaerobic bacteria; in this respect, we obtained the expected negative correlation between pH and acetic acid.

The results of sIgA obtained were varied. During the study period, the sIgA concentration decreased in both groups, with a trend towards a lower decrease in the GOS group. However, we obtained higher values than those reported for studies with GOS-/FOS-containing formulas [29] and GOS-/polydextrose (PDX)-containing formulas [30]. This suggests a protective effect on the development of a gut mucosa immune response, given that the sIgA decrease was more stable over time in the infants who consumed the GOS-containing formula, reflecting a similar behaviour to that observed in breastfed infants [29, 30].

The SCFA profile is determined by the intestinal microbiota population and depends on the feeding patterns. However, in terms of quantity, acetic acid was always the main SCFA produced in both groups of the present clinical trial. This reflects the SCFA profile of infants of the same age and is consistent with the results of other studies with GOS-/FOS-containing formulas [23, 27, 28]. The influence of GOS supplementation was shown by the significant increase in the percentage of acetic acid and the decrease in butyric acid observed, a similar pattern to that described in breastfed infants [27, 28, 31]. The faecal acetic/butyric acid profile represents events that occur in the colon and is closely related to the composition of intestinal microbiota, especially the abundance of *Bifidobacterium*, as indicated by the positive correlation that we found between acetic acid and the *Bifidobacterium* population and the negative correlation between butyric acid and *Bifidobacterium*. On the other hand, the high propionic acid content of the faeces of the control group may indicate the presence of more complex microbiota, given that propionate and butyrate are produced by *Bacteroides* and *Clostridium*, but not by *Bifidobacterium.* Several animal and in vitro assays have shown that microbial products such as SCFAs interact directly with immune cells and enterocytes and modify their activity at cell receptor level [3]. We observed a greater increase in the *Bifidobacterium* genus count during the feeding period in the GOS group compared with the control group. Results were similar to previous reports of increased *Bifidobacterium* associated with GOS-containing [13, 14] and GOS-/FOS-containing [4, 28, 32] infant formulas. A greater number of *Bifidobacterium* may be necessary for post-natal maturation of the immune system and thus have protective effects against infections and allergy by stimulating the intestinal microbiota in early stages of life.

The current study demonstrated a significant increase in the percentage of infants colonised by *B. adolescentis* at 4 months of age, unlike previous studies in which no clear difference in faecal *Bifidobacterium* species between infants receiving formulas with or without prebiotics was demonstrated [20, 30]. Our study also described a trend towards increased counts in the GOS group, whereas previous studies have only reported a diversity of species similar to that observed in breastfed infants associated with the consumption of prebiotic-containing formulas. Consistent with previous reports of prebiotic-containing formulas [4, 19, 28], no significant changes in *Lactobacillus* were observed in the current study. Regarding *C. difficile*, in our study, the percentage of infants colonised was lower in the GOS group and the bacterial count increased significantly during the feeding period, with a higher concentration in the control group at 4 months of age. Scholtens et al. [32] also found a lower percentage of *Clostridium* spp. among infants fed a GOS-/FOS-containing formula for 26 weeks. However, these findings contrast with those reported in subsequent publications that found no differences in the counts between the formula-fed groups, although there were differences between these groups and breastfed infants [30, 33].

The bowel habit is a useful marker of intestinal function, especially in the colon. It can be defined by frequency of defaecation, faecal consistency and weight and form of faeces. The typical higher frequency of defaecation and softer faeces pattern observed in breastfed infants is considered the standard of reference for comparison with formula-fed infants. In the current study, the frequency of defaecation and faecal consistency were significantly influenced by the GOS-containing formulas. Other previous studies involving prebiotic-containing infant formula found no difference in the frequency of defaecation, but faecal consistency was significantly improved, implying that the faeces became softer [30, 33]. In agreement with several previous reports with GOS-/FOS-containing formulas [34, 35], we also found a higher frequency of defaecation, especially up to the sixth month, mainly due to the introduction of complementary feeding. In addition, infants fed the GOS-containing formula showed higher prevalence of loose faeces and fewer formed/soft faeces than the infants in the control group. Other researchers have also described the softening of faeces in infants fed formulas containing GOS [13], GOS/FOS [4, 35], GOS/FOS/pectin acidic-oligosaccharides (pAOS) [36] or GOS/PDX [30, 37]. This effect is potentially beneficial in reducing the hard faeces of formula-fed infants and is probably a result of the osmotic stimulation caused by SCFAs during the fermentation of GOS by colonic bacteria.

Regarding the clinical relevance of the results, especially concerning infections and allergy, only three studies with GOS-/FOS-containing formulas have reported a reduction in infectious episodes [4–7]. In these studies, the feeding time was only 6 months, the infant formula used was hydrolysed, the infants involved had a high risk of atopy in two studies and one study was not blinded, with a wide inclusion age. In our study, infections and the number of episodes of diarrhoea and URTI showed no reduction. In fact, a systematic review has concluded that the data available in relation to the use of prebiotics in infant formulas for the prevention of infections remain inconclusive [9]. Concerning the use of antibiotics, we found no differences between the feeding groups, unlike the two previous studies with hydrolysed infant formulas [6, 7].

Neither the age of enrolment nor the GOS dose seemed to affect the differences found. In the study carried out by Arslanoglu et al. [5], the formula contained 0.8 g/dl of GOS/FOS and the enrolment age was 13 days. The authors stated that early feeding intervention had a more noticeable influence on the immune system, unlike in our study, which used lower doses (0.44 g/dl of GOS) in which the infants were older at enrolment (30 days). However, Bruzzese et al. [7] described the protective effects of a GOS-/FOS-containing formula (0.4 g/dl of GOS/FOS) when infants were even older at enrolment (54 days of age). In contrast, in a study using a GOS-/PDX-containing formula provided to infants aged 9–48 months for 108 days, Ribeiro et al. [37] observed no difference in the incidence of diarrhoea or URTI, despite recording softer faeces and a greater number of depositions in the supplemented group, as was the case in our study. Therefore, the effects of doses and age of inclusion are not conclusive and need further research.

Recent studies have described how feeding interventions that enhance microbial diversity in early life may provide an effective way of preventing eczema in high-risk infants [38]. Moro et al. [4] reported a significant reduction in eczema in infants up to 6 months at high risk of atopy and fed a hydrolysed GOS-/FOS-containing formula. The same working group confirmed the protective effect beyond the feeding period, over the first 2 years of life, in a study with 134 infants [6]. Grüber et al. [8] enrolled infants under 8 weeks and with low risk of atopy who were allocated to a control or prebiotic groups with a GOS-/FOS-/pAOS-containing formula. The researchers reported a significant reduction in AD in the prebiotic group up to the first year. However, the severity of AD was not significantly affected, and there were no statistically significant differences in allergic sensitisation to hen’s egg or cow’s milk between the groups [8]. Further evidence supporting the use of prebiotics in infant formulas to prevent allergy is required, according to two recent systematic reviews [9, 10].

The infants in the present study were all healthy, term infants who lived in a modern environment with a low risk of infectious disease. Moreover, both groups were fed a formula containing nucleotides and long-chain polyunsaturated fatty acids, substances that have also been said to be involved in immune system regulation [39, 40], the only difference being the inclusion (or not) of GOS. Despite the absence of a significant difference between the two groups in terms of clinical manifestations of either infections or allergies, differences in key leading indicators of immunity were found in association with a demonstrated prebiotic effect. In the present study, infections and allergy manifestations were infrequent both in the GOS group and in the control group, making it difficult to demonstrate a reduction from an already low baseline incidence. Further studies in populations with a higher incidence of infections, perhaps in developing countries, and on the effects on allergy manifestation in infants with a high risk of atopy are needed.

Our study did not include a group of breastfed infants. Nevertheless, our results were compared with the data currently available in the scientific literature on breastfed outcomes.

We conclude that the feeding of a GOS-containing infant formula produced a definite prebiotic effect, consisting of changes in faecal composition and microbiota, and in faecal consistency and frequency of defaecation. We observed no reduction in the incidence of infections or allergic manifestations during the first year of life. The critical association between prebiotic supplementation and improved immunological function in healthy human infants is still to be fully defined. Further trials, potentially in at-risk groups, are needed to verify whether adding prebiotics to infant formulas has a preventive effect on the infant immune system, as well as to elucidate the specific mechanisms of action.
